# Effects of Surfactants on the Degradation of Diclofenac by Manganese Oxide

**DOI:** 10.3390/ijerph17124513

**Published:** 2020-06-23

**Authors:** Wen-Hui Kuan, Yu-Jung Liu, Ching-Yao Hu

**Affiliations:** 1 Department of Environmental and Safety Engineering, Ming Chi University of Technology, Taishan, New Taipei City 24301, Taiwan; whkuan@mail.mcut.edu.tw; 2 Chronic Disease and Health Promotion Research Center, Chang Gung University of Science and Technology, Chiayi 61363, Taiwan; 3 Graduate Institute of Environmental Engineering, National Taiwan University, 71 Chou-Shan Rd., Taipei 10617, Taiwan; yujungliu77@gmail.com; 4 School of Public Health, Taipei Medical University, 250 Wu-Xing Street, Taipei 11031, Taiwan

**Keywords:** manganese dioxide, diclofenac, anionic surfactant, cationic surfactant

## Abstract

Amine-containing pharmaceuticals are the most often detected pharmaceuticals in wastewater and ambient aquatic environments. They can usually be degraded by manganese oxide (MnO_2_), which is a common natural oxidant in soils. Surfactants often coexist with pharmaceuticals in wastewater. Some amine-containing pharmaceuticals, such as diclofenac (DIC), are acidic and are thus ionic compounds in neutral conditions. These compounds, therefore, have similar properties to surfactants. Surfactants, thus, may influence the adsorption and degradation processes of DIC by MnO_2_. The effect of the type of surfactant on the degradation of DIC by MnO_2_ was investigated in this study with the addition of two common biodegradable surfactants (cetyltrimethyl-ammonium bromide (CTAB) and sodium dodecylsulfate (SDS)). The results indicated that the cationic surfactant (CTAB) significantly increased the degradation rate in neutral and alkaline conditions. On the other hand, the anionic surfactant (SDS) slightly increased the DIC removal rate in an acidic condition but significantly decreased the removal in neutral and alkaline conditions. Coexisting cationic surfactants not only influenced the kinetics but also altered the transformation mechanism of DIC by MnO_2_. Decarboxylation is the main transformation mechanism of DIC in the presence of CTAB, while both decarboxylation and hydroxylation are the main transformation mechanisms in the absence of CTAB.

## 1. Introduction

Diclofenac (2-((2,6-dichlorophenyl) amino) phenylacetic acid, DIC) is one of the most widely used non-steroidal anti-inflammatory drugs (NSADs) in the world. It is also an amine-containing pharmaceutical and can evade wastewater treatment because of its high hydrophilic nature [1] and low biodegradability [2]. Therefore, it is found in many aquatic environments [3,4]. It may be toxic to aquatic organisms and harmful to embryos, infants, children, and adults with weak constitutions and those who are sensitive to pharmaceuticals [5,6,7,8]. Moreover, continuous exposure to low concentrations of the pharmaceutical may cause unexpected health risks to humans and other organisms. The bioaccumulation and sublethal effects of DIC were observed in rainbow trout with the lowest observed effect concentration (LOEC/28 days of 1.0 µg L^−1^) in the range of discharge levels [9,10].

Manganese dioxide (MnO_2_) is an effective natural oxidant of organic pollutants, including phenols [11], chlorophenol [11,12,13,14], and aliphatic amines and anilines [13,15,16], in soils and sediments. More recently, MnO_2_ was used to remove antibacterials and related compounds with phenolic and fluoroquinolonic moieties [17,18], aromatic *N*-oxides [19], tetracyclines [20], and estrogenic compounds such as the synthetic hormone 17R-ethinylestradiol [21,22]. A mechanism involving the sorption of compounds onto the oxide surface and subsequent electron transfer was proposed for oxidation reactions. Forrez et al. [23] used chemically and biologically produced manganese oxides to oxidize DIC. They found that both chemical and biological MnO_2_ could efficiently oxidize DIC in an acidic condition (pH 4.7), but the removal rate dramatically decreased at higher pH values. The decrease in the removal rate at higher pH values may be partly explained by a decrease in the adsorption rate due to an increase in the surface charge of MnO_2_ and a decrease in its oxidation power due to a lower proton concentration, as shown in the following reaction [23]:

(1) $$MnO_{2}+4H^{+}+2e^{–}\to Mn^{2+}+2H_{2}O$$

Huguet and coworkers [24] used a MnO_2_ filter to eliminate DIC. They also found that the reaction rate decreased as the pH increased and the ionic strength decreased. Both of these studies found hydroxyl-diclofenac and 5-iminoquinone DCF to be the main products, and, therefore, both hydroxylation and decarboxylation are the main mechanisms for DIC degradation by MnO_2_.

Some personal care products, such as shower gels and shampoos, commonly contain a lot of surfactants to stabilize the emulsions. Surfactants from these products frequently occur, along with pharmaceuticals, in wastewater. Both kinds of compounds can evade biotreatment processes and escape into the environment [25,26]. Since surfactants can significantly influence the fate and transport of numerous organic contaminants [27,28,29,30,31,32], it is also possible that they could influence the degradation of DIC by MnO_2_.

In this study, we investigated the effects of surfactants on the degradation of DIC by MnO_2_. Two common biodegradable surfactants, cetyltrimethyl-ammonium bromide (CTAB) and sodium dodecylsulfate (SDS), were used to respectively compare the effects of cationic and anodic surfactants. The effects of the pH levels and dosages of the surfactants and MnO_2_ were investigated. Degradation products were also identified by ultra-performance quadrupole time of flight mass spectroscopy (UPLC-Q-TOF-MS) to compare the effects of these two surfactants on the degradation mechanism.

## 2. Experimental Section

### 2.1. Materials

All chemicals used in this study were of analytical grade and were purchased from Sigma-Aldrich (St. Louis, MO, USA), J. T. Baker (Phillipsburg, NJ, USA), and Riedel-deHean (Seelze, Germany). Stock solutions of DIC sodium (10 mM), SDS (10 mM) and CTAB (10mM) were prepared by adding suitable amounts of these chemicals into deionized water. Ten grams of MnO_2_ powder purchased from Tosoh was suspended in a 1 L solution to form a 10 g L^−1^ stock solution. 

### 2.2. Batch Experiments

Experiments were conducted at different pH values (pH 4–9). For each batch system, various amounts of MnO_2_ were added to 15 ml glass centrifuge tubes. NaH_2_PO_4_ at 0.005 M and NaH_2_BO_3_ were added to the solution as buffers. Various proportions of 0.1 M HCl and NaOH were used for pH adjustment. A stock DIC solution was added to a tube to obtain an initial concentration of 0.1 mM. The centrifuge tubes were covered with aluminum foil to prevent exposure to light. The suspensions were equilibrated at 25 °C by end-over-end rotation at 10 rpm for 24 h. All experiments were conducted in duplicate. Controls (no MnO_2_ powder) were also established, using a similar preparatory process, to account for sorption onto the glass tubes and other reactions in the solutions.

### 2.3. Sample Preparation and Analysis

An Orion-2101 pH meter (Columbus, Ohio, USA) was used to measure the pH. Suspensions were centrifuged at 8000 rpm for 40 min in a centrifuge (Pico 17, Thermo Scientific, Waltham, MA, USA), and the supernatant was quantified using high-performance liquid chromatography (HPLC) (L-7200, Hitachi, Japan) with a diode array detector (DAD) (L-7455, Hitachi). Chromatographic separation was done using a C_18_ column (RP-18 GP 150 × 4.6 mm, 5 µm, Mightysil) with an eluent consisting of 60% acetonitrile and 40% acidified water (25 mM phosphoric acid). UV detection was performed at 270 nm. The flow rate was 1.0 mL min^−1^, and the injection volume was 20 μL. The zeta potential of the particles was measured using an electroacoustic spectrometer (DT-1200, Dispersion Technology).

### 2.4. Identification of Oxidation Products

The first screening of the major oxidation products was performed with UPLC-MS. The system consisted of an Agilent 1100 Series LC (Agilent, Palo Alto, CA, USA) with a CTC PAL auto-sampler (CTC Analytica, Carrboro, NC, USA) separation module, interfaced with an API 4000 triple quadrupole mass spectrometer (Applied Biosystems AB/MDS Sciex, Foster City, CA, USA). The LC column was a Luna Polar RP (150 × 2.1 mm internal diameter) column that was purchased from Phenomenex (Torrance, CA, USA). The HPLC gradient was established by mixing two mobile phases: acetonitrile and deionized water with 10 mM formic acid. Chromatographic separation was achieved with the following gradient: 0–1 min: 0% acetonitrile; 1–5.0 min: linear gradient to 100% acetonitrile; 5.0–10 min: 100% acetonitrile; 10–10.1 min: back to 0% acetonitrile; and 10.1–15 min: 0% acetonitrile. The flow rate was 0.5 mL min^−1^, and the injection volume was 10 μL. The parameters of the mass spectrometer which was operated in both positive and negative ion mode were a curtain gas pressure of 20 psi, ion source gas 1 pressure of 30 psi, ion source gas 2 pressure of 40 psi, source temperature of 500 °C, declustering potential of 105 V, entrance potential of 10 V, nebulizer current of 5 μA, and the interface heater was switched on. Positive and negative ions were scanned in the range of 100~500 m/z at a cycle time of 1 s. The data obtained were processed with Analyst 1.4.2 software (Framingham, MA, USA).

## 3. Results and Discussion

### 3.1. Effects of the Types of Surfactants on DIC Oxidation by MnO_2_

The X-ray powder diffraction pattern of the MnO_2_ material (Figure S1) displays a 1 × 1 molecular sieve structured pyrolusite (JCPDS 24-0735) with characteristic reflections (angle position 2θ = 37.3°, 42.8°, 56.7° and 67.9°), and the peak broadness is indicative of the small crystal size. The average diameter of MnO_2_ as calculated using the Scherrer formula is approximately 156 nm, as shown in Figure S2. The Brunauer–Emmett–Teller (BET) specific surface area of the MnO_2_ was 45.6 m^2^·g^−1^.

**Figure 1:**
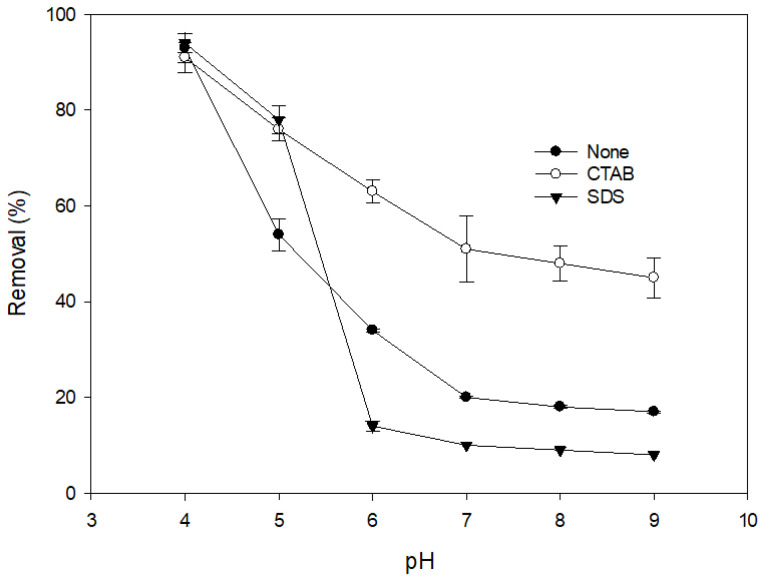
Effect of anionic and cationic surfactants on the removal efficiency of diclofenac (DIC) by manganese oxide. (reaction time = 1 day, (MnO_2_) = 100 mg L^−1^, initial DIC conc. = 100 µM, surfactant conc. = 100 µM, T = 25 °C).

**Figure 2:**
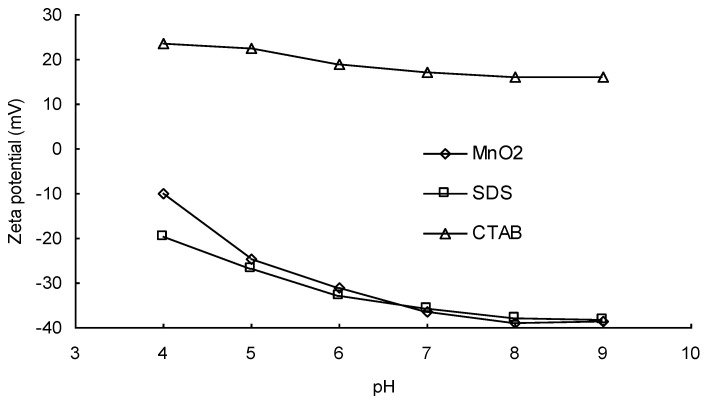
Variations in the zeta potential on the manganese oxide powder surface at different pH values and effects of the addition of different kinds of surfactant.

Figure 1 shows the effects of different types of surfactant on the removal of DIC by MnO_2_. It is notable that the addition of a cationic surfactant (CTAB) significantly improved the removal of DIC at all pH values. Figure 2 shows variations in the zeta potential of MnO_2_ at different pH values and the effects of the addition of different kinds of surfactant. It is notable that after the addition of a cationic surfactant, the zeta potential remained positive even at a high pH. The positively charged surface should have had a higher affinity for anions. After ionization, the structure of an acidic pharmaceutical molecule such as DIC is similar to that of an anionic surfactant, containing a hydrophilic head and a hydrophobic tail. Therefore, when cationic surfactant molecules are adsorbed onto the surface of a metal oxide, the anionic DIC ions can also be adsorbed because of attractive forces between the hydrophobic tails of the two molecules. Heri and co-workers [27] found that a cationic surfactant can enhance the adsorption of acidic pharmaceuticals onto mineral materials in a high pH condition. They stated that ion pairing between the anionic pharmaceutical and the cationic surfactant might be formed and might reduce the solubility of DIC, thereby increasing the driving force for adsorption. A similar effect may also exist between DIC and CTAB. The addition of a cationic surfactant, therefore, could enhance the reaction efficiency because of the increase in the mass diffusion rate due to the rise in DIC adsorption onto MnO_2_.

The presence of an anionic surfactant (SDS) only improved the removal of DIC in an acidic condition and decreased the removal significantly when the pH exceeded 6. The pKa of DIC was 4.15 [33], which means there was some undissociated DIC in the acidic condition. The addition of an anionic surfactant can form a surface micelle on the mineral surface. This micelle has a high affinity with the undissociated DIC. The addition of SDS, therefore, enhanced the removal of DIC in an acidic condition. At higher pH values, the dissociated DIC was repelled by the negative surface. The anionic surfactant may have competed with the DIC to be adsorbed onto active sites on the MnO_2_. The removal of DIC, therefore, significantly decreased. Most of the surfactants in wastewater are anionic surfactants. This phenomenon indicates that the removal of DIC by MnO_2_ may be inhibited by anionic surfactants during the treatment of actual wastewater.

### 3.2. Effects of the Dosages of MnO_2_ and CTAB

**Figure 3:**
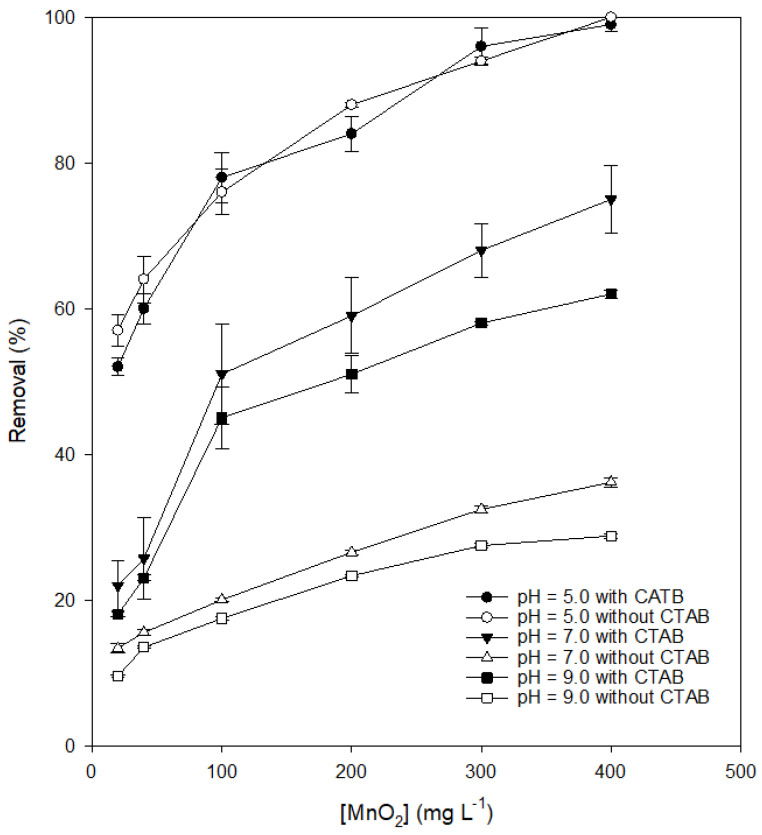
Effect of the dosage of MnO_2_ on the removal of diclofenac (DIC) in the presence and absence of cetyltrimethyl-ammonium bromide (CTAB). (Reaction time = 1 day, initial DIC conc. = 100 µM, surfactant conc. = 100 µM, T = 25 °C).

Figure 3 shows variations in DIC removal with the dose of MnO_2_ at different pH values in the presence and absence of CTAB. The removal of DIC increased as the dosage of MnO_2_ exceeded 100 mg/L with the addition of CTAB. If the dosage was not high enough (<100 mg L^−1^), enhancement significantly dropped. This phenomenon should have been because adding CTAB can only increase the adsorption rate of DIC onto the MnO_2_ surface but cannot increase the electron transformation rate. The determining step should be adsorption if the dose of MnO_2_ is high enough. Otherwise, the determining step should be electron transformation. This means that the effect of coexisting surfactants was insignificant if the dosage of oxidants was not high enough. The addition of CTAB improved the adsorption capacity but could not accelerate the electron transfer rate. If the dose of MnO_2_ decreased, the increase of the adsorption of DIC would be limited.

**Figure 4:**
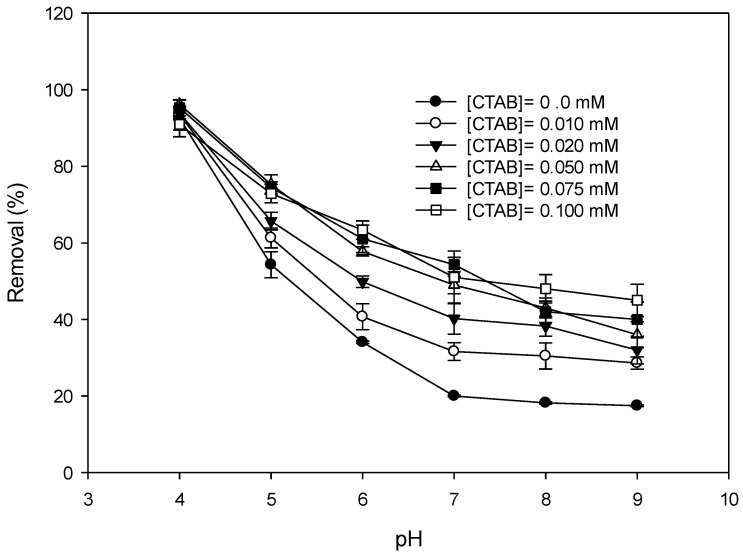
Effect of the dosage of CTAB on the removal of diclofenac (DIC) by MnO_2_. (Reaction time = 1 day, initial DIC conc. = 100 µM, (MnO_2_) = 100 mg L^−1^, T = 25 °C).

Figure 4 shows variations in the DIC removal by MnO_2_ at various concentrations of CTAB. Removal increased with an increase in the dosage of CTAB if the concentration of CTAB was low. When the CTAB concentration exceeded 50 µM, the increase in CTAB seemed unable to improve the DIC removal by MnO_2_. This fact should have been due to the formation of a CTAB layer on the surface of the MnO_2_. Ionized surfactants are adsorbed onto an oppositely charged (metal oxide) surface through their polar moieties. At higher concentrations, a monolayer of surfactant forms on the surface. The hydrophilic head groups are, thus, in contact with the metal oxide surface, while the hydrophobic tails are in contact with the solution. This creates a hydrophobic surface, leading to the further adsorption of surfactants through their hydrophobic components, thereby forming surface micelles. The adsorption of surfactants onto the oppositely charged surface significantly increases with an increasing surfactant concentration until a surface micelle forms [34]. The formation of a surface micelle, however, may hinder electron transformation. The removal of the DIC, therefore, did not increase when the concentration of CTAB was too high.

### 3.3. Effects of CTAB on the Kinetics of Degradation

**Figure 5:**
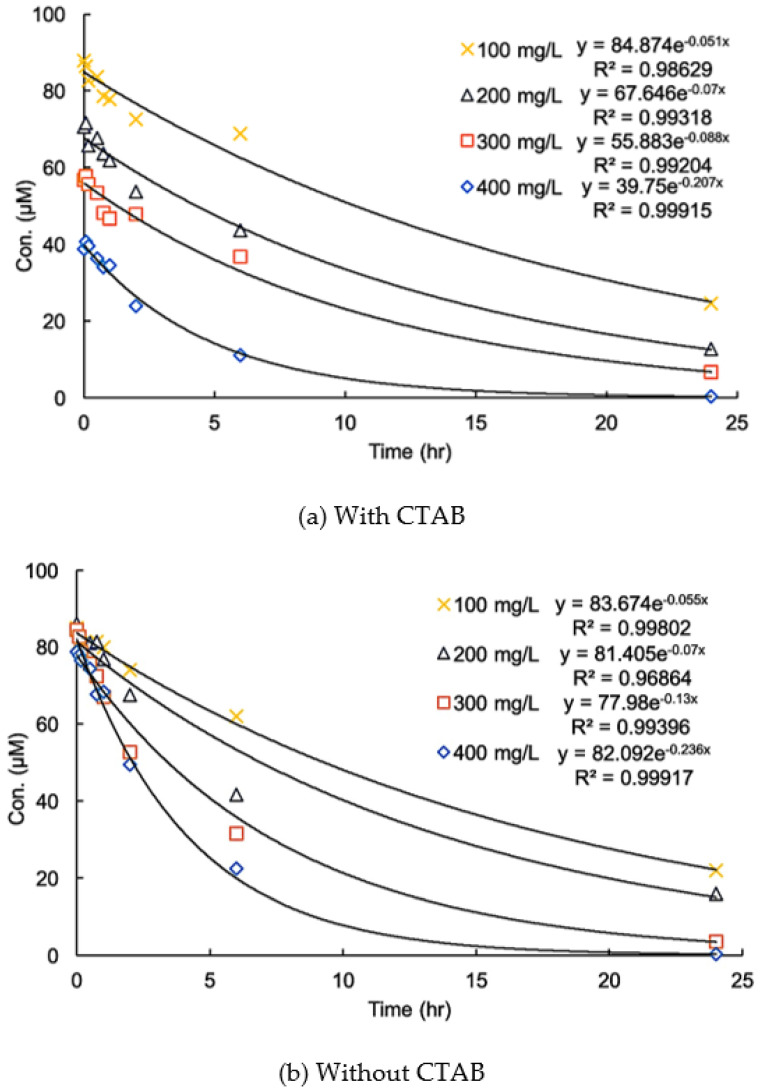
Effect of CTAB on the kinetics of the degradation of diclofenac (DIC) with different dosages of MnO_2_ at pH 5.0 (a) with CTAB and (b) without CTAB (initial DIC conc. = 100 µM, (CTAB) = 100 µM, T = 25 °C).

Figure 5 shows variations in the time of the residual DIC concentration in systems in the absence and presence of CTAB. The pseudo-first-order kinetic constant (*k*) was calculated by the following equation:

(2) $${\left[DCF\right]}_{t}={\left[DCF\right]}_{0}e^{-kt}$$

where *t* is the reaction time. The equation fit the experimental data very well in acidic conditions, but the initial DIC concentration in the presence of CTAB decreased with an increase in the MnO_2_ dosage. 

This fact indicates that the addition of CTAB did not alter the reaction kinetics but significantly increased the adsorption of DIC on the surface of MnO_2_. This result confirms the inference that the addition of a cationic surfactant can enhance the reaction rate by raising the mass diffusion rate of adsorption.

It was noted that the pseudo-first-order kinetic model cannot be used for DIC degradation in neutral and alkaline conditions. The concentration of DIC did not decrease until the reaction time exceeded 1 day in the presence of CTAB. This phenomenon indicates that the higher removal of DIC was mainly due to higher adsorption and implies that electron transfer between DIC and MnO_2_ was limited in the early reaction period. This fact should have been due to the structure of the surface micelles, which are comprised of a double layer. As mentioned earlier, the surface of MnO_2_ is negatively charged in a neutral condition. The inner layer of the micelles, therefore, should mainly consist of CTAB, while DIC ions should mainly be in the outer layer, and a period of time is needed for them to diffuse to the inner layer. The transformation of DIC was limited in the early period of the reaction.

### 3.4. Effects of CTAB on the Products

The chromatograms and mass spectra of the products of DIC degradation by MnO_2_ in the presence and absence of CTAB are provided in the “Supplementary Data” (Figures S3–S8). Due to the ionic nature of DIC, two ionization methods, ESI^+^ and ESI^−^, were used to determine the degradation products, and the results are listed in Table 1 (without CTAB) and Table 2 (with CTAB). Four products in the absence of CTAB were found. Two of them were reported in the literature. P_1(A)_ (m/z = 346) should be tri-hydroxyl-DIC, as reported by Monteagudo et al. (2018) and Yu et al. [35,36]. I_2(A)_ (m/z = 308) should be 5-iminoquinone DCF, as reported by Forrez and coauthors [23]. There are two new products: U_1(A)_ (m/z = 298) should be an oxidized-decarboxylated DIC and U_2(A)_ (m/z = 603) should be a dimer of DIC (m/z = 295) and hydroxy-DIC (m/z = 309). The same result was also reported by Huguet and coauthors [24]. This finding reveals that polymerization or dimerization may occur during DIC degradation by MnO_2_, as with other aromatic amines and phenolic compounds [37,38,39]. The other new intermediate, U_2(A)_ (m/z = 298), should be an oxidized-decarboxylated DIC. The two new intermediates were only detected in the positive mode, while previous studies usually use the negative mode to detect the products of DIC. This might be the reason that they did not detect them previously.

Three products were found in the presence of CTAB in an acidic condition. P_1(B)_ (m/z = 283) should be a decarboxylated derivative of DCF, which was reported by Huguet and coauthors [24]. P_2(B)_ (m/z = 266) and P_3(B)_ (m/z = 250) should be other decarboxylated derivatives of DCF, as reported by Liu et al. [40] and Martínez et al. [41]. It is notable that no quinone was detected, and decarboxylation, therefore, seemed to be the major reaction in the presence of CTAB. This phenomenon can also be explained by the formation of surface micelles. Only the ionic moiety can contact the MnO_2_ surface because of the arrangement of the molecules in the micelles. The aromatic ring cannot attach to the surface of MnO_2_. Decarboxylation, therefore, was the major reaction. A similar phenomenon has also been found for the decarboxylation of another anionic compound (6-Nitrobenzisoxazole-3-carboxylate) [42].

No product was found in neutral condition in the early reaction period. The decarboxylated derivative of DCF (P_1(B)_, m/z = 283) was found after 1 day. The other products, however, were not detected. This fact confirms the inference that the transformation of DIC was limited in the early period of the reaction.

## 4. Conclusions

The results of this study reveal two major findings. Firstly, the presence of different kinds of surfactants significantly influenced the oxidation of DIC by MnO_2_. The presence of a cationic surfactant (CTAB) sped up the reaction rate by increasing the adsorption capacity in neutral and alkaline conditions. On the other hand, the anionic surfactant increased the DIC removal rate in an acidic condition but decreased removal in neutral and alkaline conditions. Secondly, the addition of a cationic surfactant may have altered the reaction mechanism of DIC removal by MnO_2_. Both hydroxylation and decarboxylation were the main reactions in the absence of a cationic surfactant. Diclofenac-2,5-iminoquinone might not form in the presence of a cationic surfactant, which means that decarboxylation was the main reaction.

## Figures and Tables

**Table 1:** MS (mass spectroscopy) data of products in the absence of CTAB.

Compound	RT (min)	Mode	m/z	Possible Structure	Reference
DIC	3.18	ESI^+^	296/298 (3/2) 278/280, 250/252, 215/217		
P_1(A)_	2.09	ESI^+^	346/348 (3/2) 328/330, 284/286, 244/246, 162/164	Tri- or dihydroxyl DIC	(Yu et al., 2013) [36]
U_1(A)_	2.48	ESI^+^ ESI^−^	298/300 (3/2) 267, 244 266/264 (3/2) 228/230	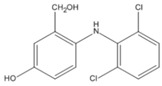	
U_2(A)_	2.94	ESI^+^	597/599 (3/4) 575/577 (3/4), 551/553 (3/4), 507/509 (3/4), 308, 267, 255	DCF+*m/z* 308 dimer or DCF+5OH-DCF dimer	
P_2(A)_	3.05	ESI^+^ ESI^−^	308, 267 283	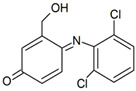	

RT, retention time; DIC, diclofenac.

**Table 2:** MS data of products in the presence of CTAB.

Product	RT (min)	Mode	m/z	Possible Structure	Reference
DIC	3.18	ESI^+^	296/298 (3/2) 278/280, 250/252, 215/217		
P_1(B)_	3.66	ESI^+^	284	Decarboxy-DIC	
P_2(B)_	2.34	ESI^−^	266 228	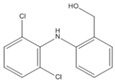	[40,41]
P_3(B)_	2.83	ESI^−^	250/252 (3/2)	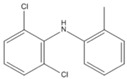	(Martínez et al., 2011) [41]
				or	
				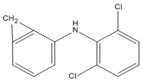	

RT, retention time; DIC, diclofenac.

## Supplementary Materials

The following are available online at https://www.mdpi.com/1660-4601/17/12/4513/s1, Figure S1: X-ray diffractometer (XRD, PANalytical X’Pert Pro MRD diffractometer) pattern of pyrolusite (JCPDS 24-0735) with characteristic reflections at 2θ of 37.3, 42.8, 56.7., Figure S2: TEM images of MnO_2_ material, Figure S3: LC/MS (ESI+) chromatographic patterns of the diclofenac (DIC) standard, Figure S4: LC/MS (ESI−) chromatographic patterns of the diclofenac (DIC) standard, Figure S5: LC/MS (ESI+) chromatographic patterns of degradation intermediates in the absence of CTAB. (pH 5.0, reaction time = 2 h, [MnO_2_]0 = 400 mg L^−1^), Figure S6: LC/MS (ESI−) chromatographic patterns of degradation intermediates in the absence of CTAB. (pH 5.0, reaction time = 2 h, [MnO_2_]0 = 400 mg L^−1^), Figure S7: LC/MS (ESI+) chromatographic patterns of degradation intermediates in the presence of CTAB. (pH 5.0, reaction time = 2 h, [MnO_2_]0 = 400 mg L^−1^), Figure S8: LC/MS (ESI−) chromatographic patterns of degradation intermediates in the presence of CTAB. (pH 5.0, reaction time = 2 h, [MnO_2_]0 = 400 mg L^−1^).
