# Supplementary material for: Effects of Surfactants on the Degradation of Diclofenac by Manganese Oxide
Source: Int J Environ Res Public Health. 2020 Jun 23;17(12):4513. doi: 10.3390/ijerph17124513 (PMC7345797; doi:10.3390/ijerph17124513)
Supplement: Supplementary file 1 [file ijerph-17-04513-s001.pdf]

Supporting Information

Effects of Surfactants on the Degradation of Diclofenac by Manganese Oxide

Wen-Hui Kuan[1, 2], Yu-Jung Liu[3] and Ching-Yao Hu[4*]

[1] Department of Environmental and Safety Engineering, Ming Chi University of Technology, Taishan, New Taipei City 243, Taiwan, ROC.

[2] Chronic Disease and Health Promotion Research Center, Chang Gung University of Science and Technology, Chiayi 61363, Taiwan, ROC.

[3] Graduate Institute of Environmental Engineering, National Taiwan University, 71 Chou-Shan Rd., Taipei 106, Taiwan, ROC.

[4] School of Public Health, Taipei Medical University, 250 Wu-Xing Street, Taipei 11031, Taiwan, ROC.

* Correspondence: School of Public Health Taipei Medical University 250 Wu-Xing Street, Taipei 11031, Taiwan, ROC. TEL: 886-2-27361661 ext. 6514; Fax: 886-2-27384831; E-mail: cyhu@tmu.edu.tw.

Contents

8 Pages

8 Figures

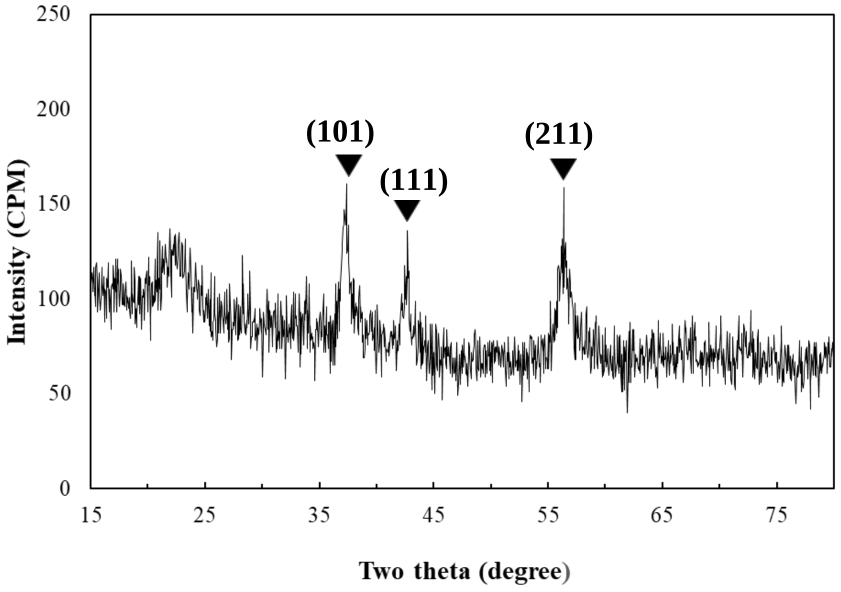

Figure S1. X-ray diffractometer (XRD, PANalytical X'Pert Pro MRD diffractometer) pattern of pyrolusite (JCPDS 24-0735) with characteristic reflections at 2θ of 37.3, 42.8, 56.7.

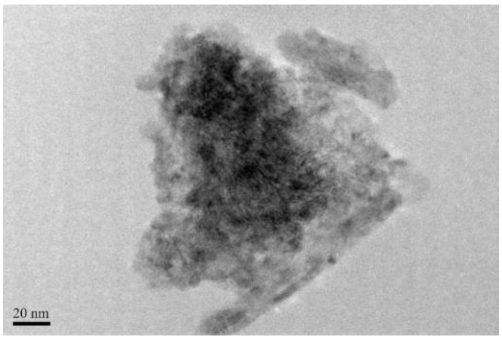

Figure S2. TEM images of MnO_2 material

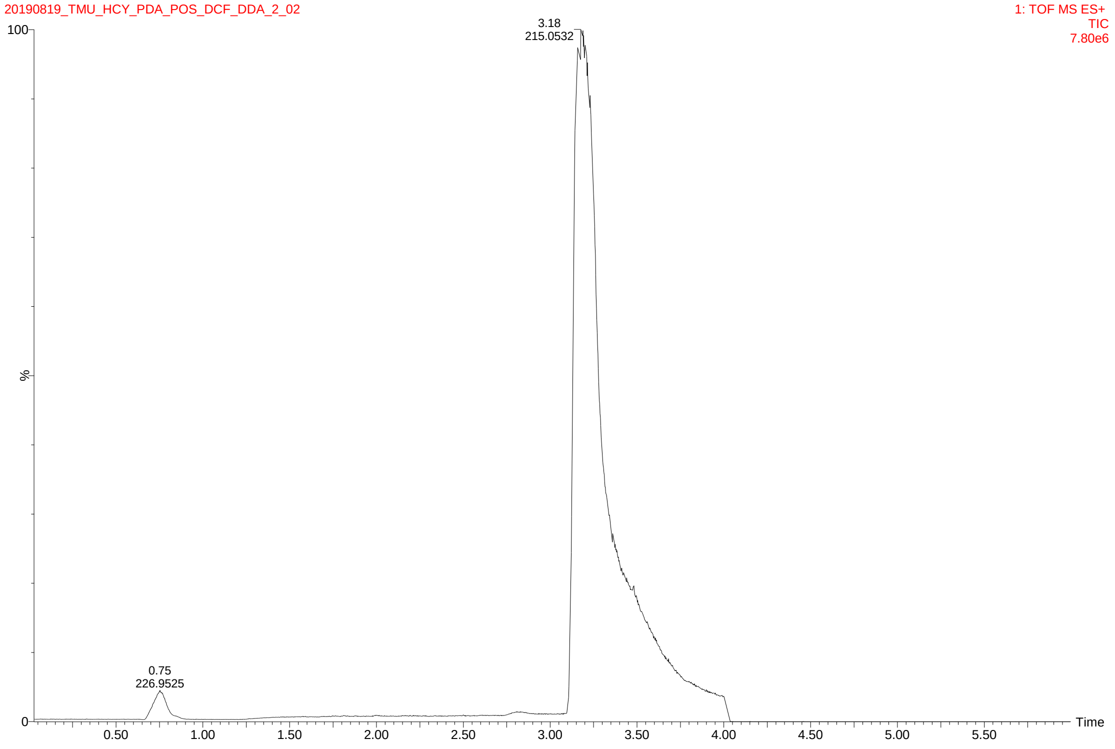

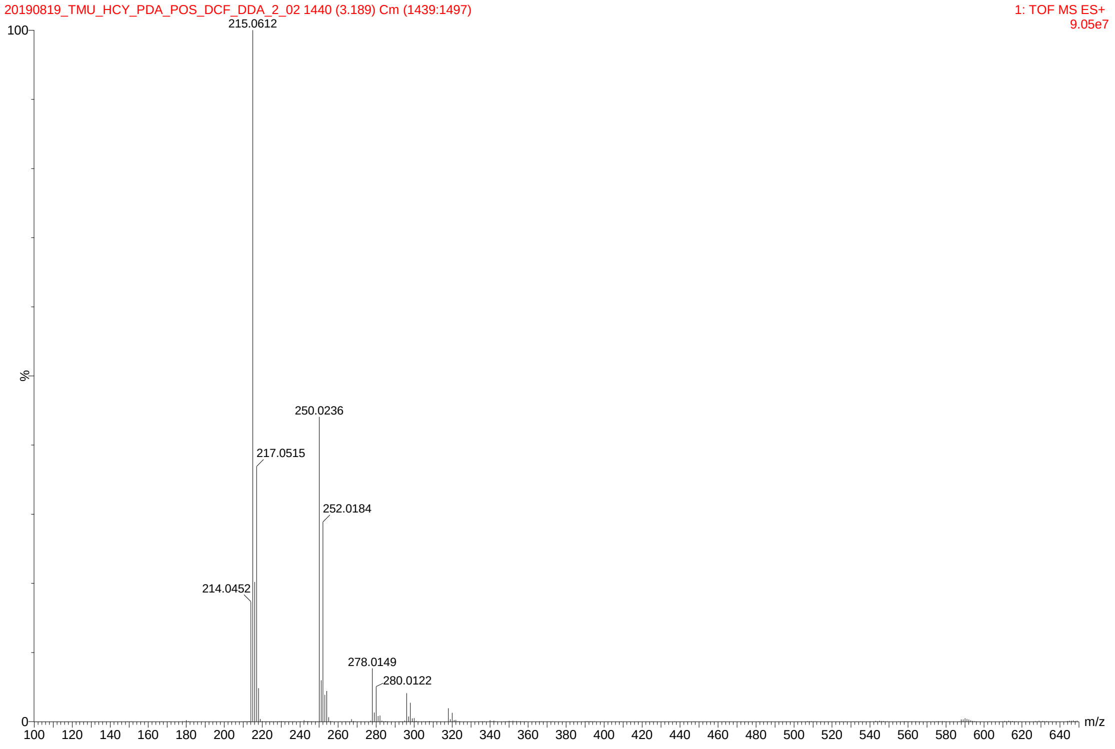

Figure S3. LC/MS (ESI^+) chromatographic patterns of the diclofenac (DIC) standard.

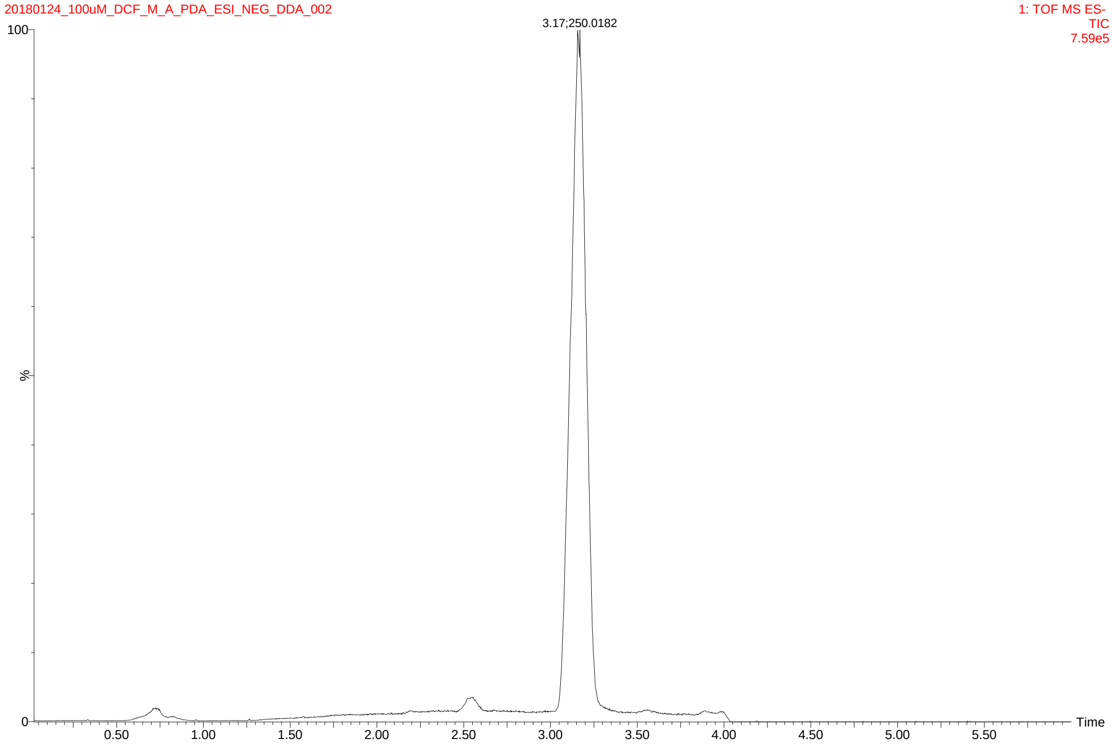

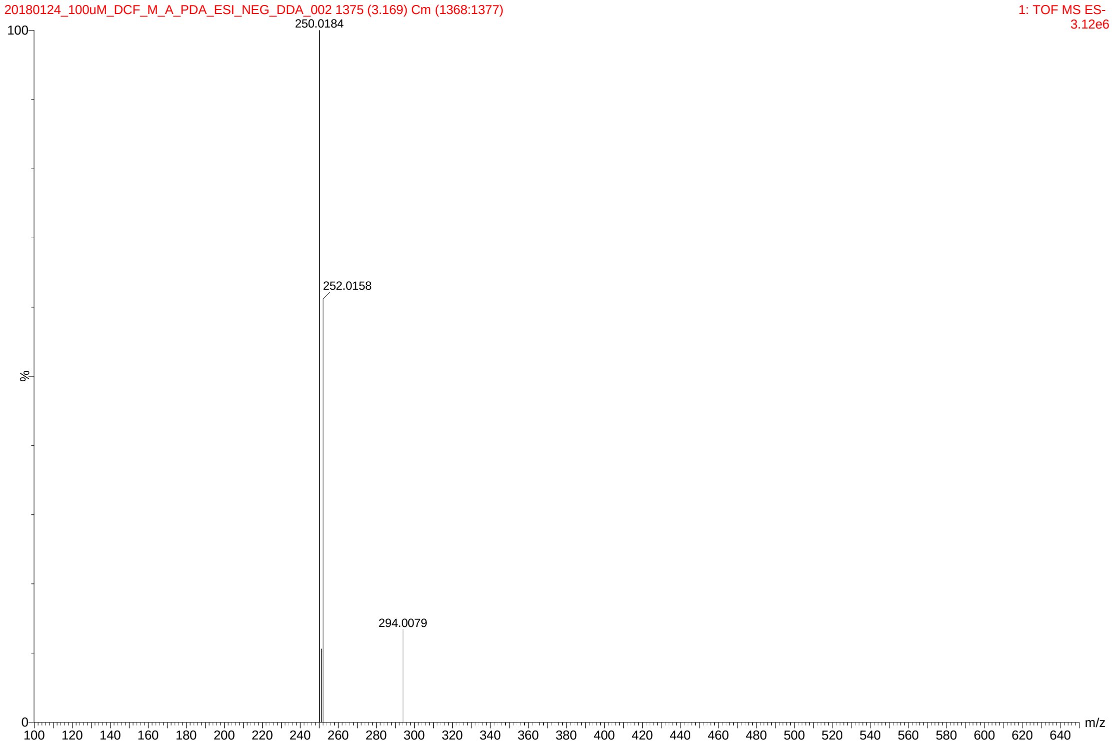

Figure S4. LC/MS (ESI$^-$) chromatographic patterns of the diclofenac (DIC) standard.

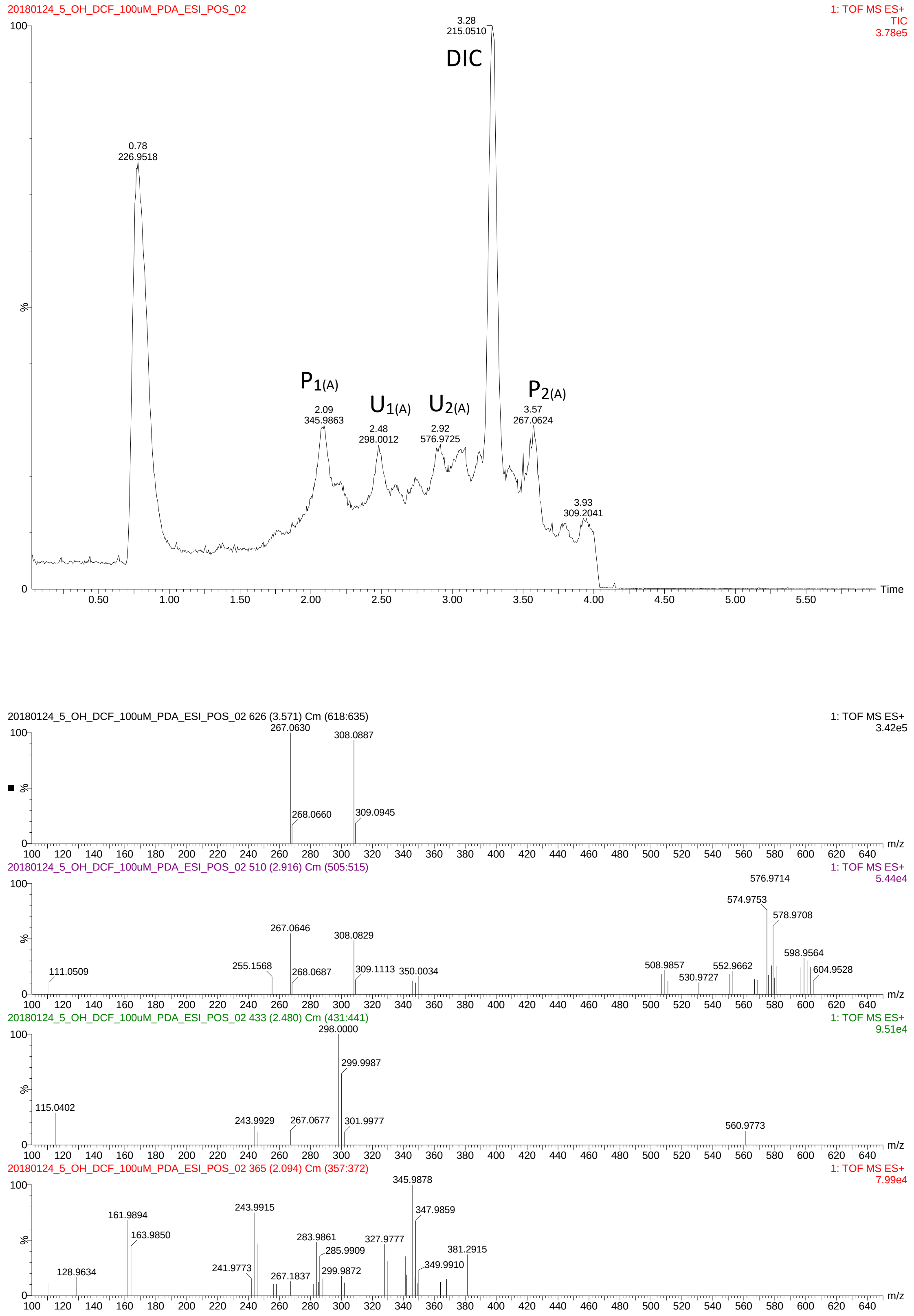

Figure S5. LC/MS (ESI^+) chromatographic patterns of degradation intermediates in the absence of CTAB. (pH 5.0, reaction time = 2 h, $[MnO_2]_0$ = 400 mg L^{-1}).

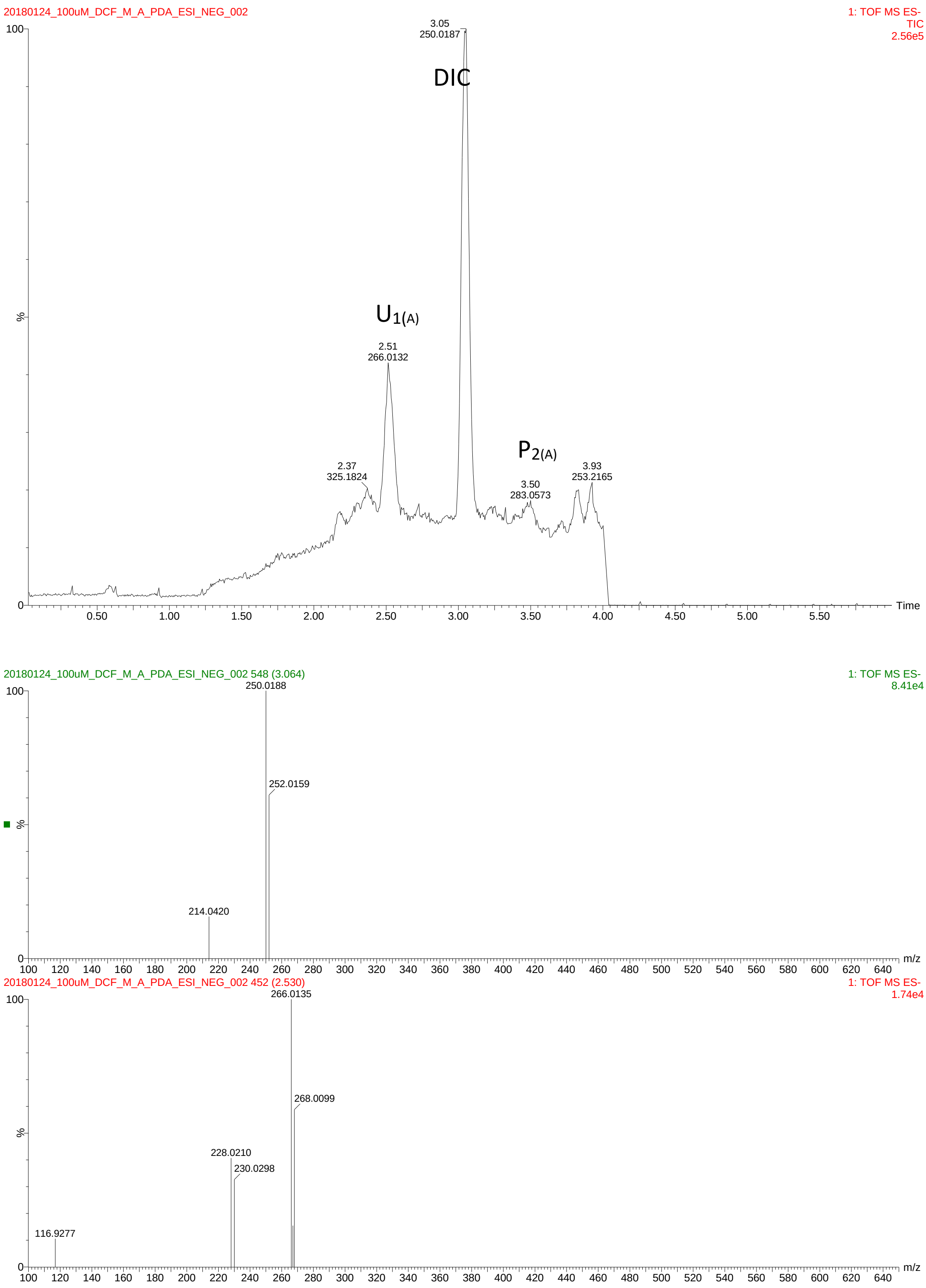

Figure S6. LC/MS (ESI$^-$) chromatographic patterns of degradation intermediates in the absence of CTAB. (pH 5.0, reaction time = 2 h, $[MnO_2]_0$ = 400 mg L^{-1}).

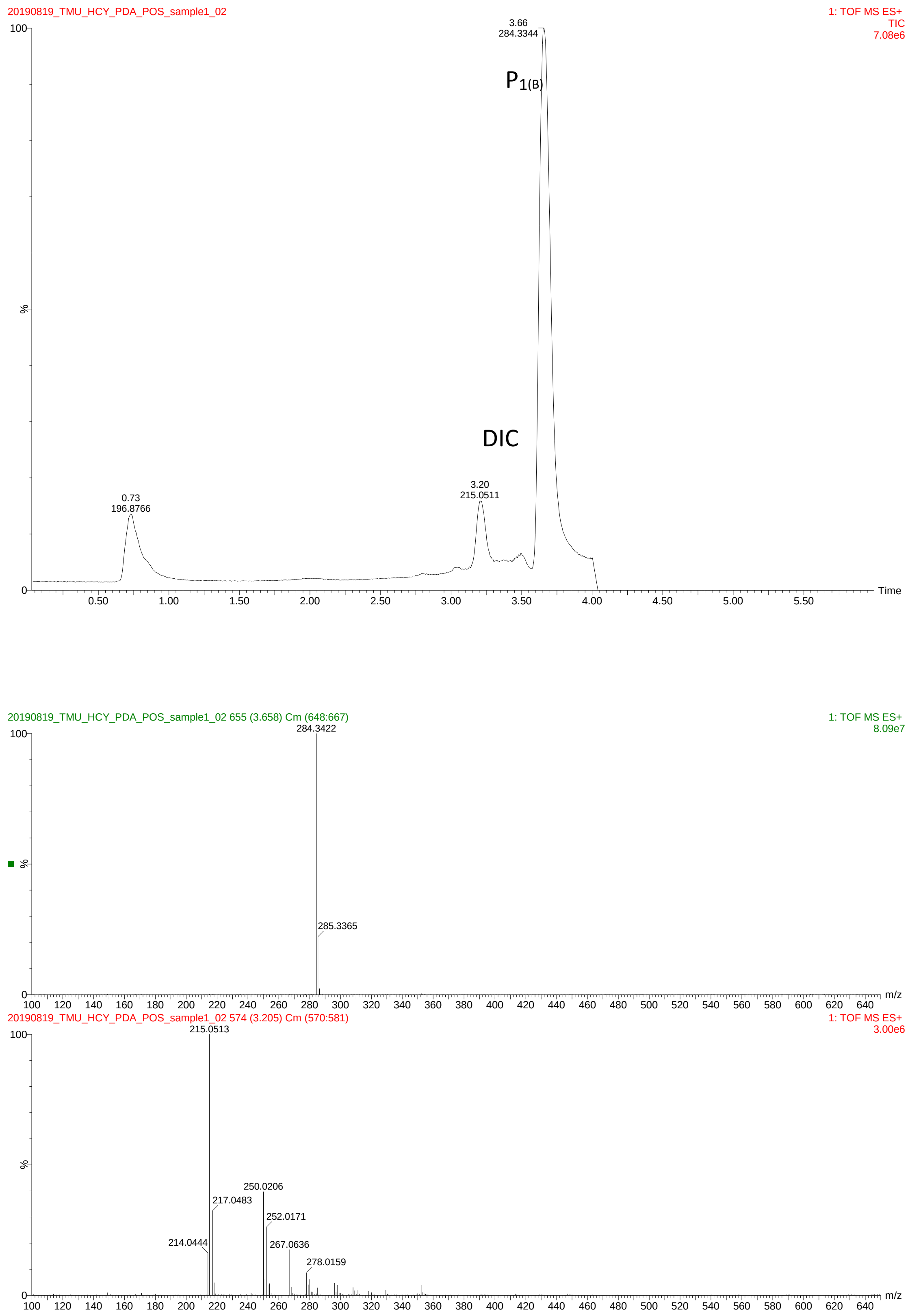

Figure S7. LC/MS (ESI^+) chromatographic patterns of degradation intermediates in the presence of CTAB. (pH 5.0, reaction time = 2 h, $[MnO_2]_0$ = 400 mg L^{-1}).

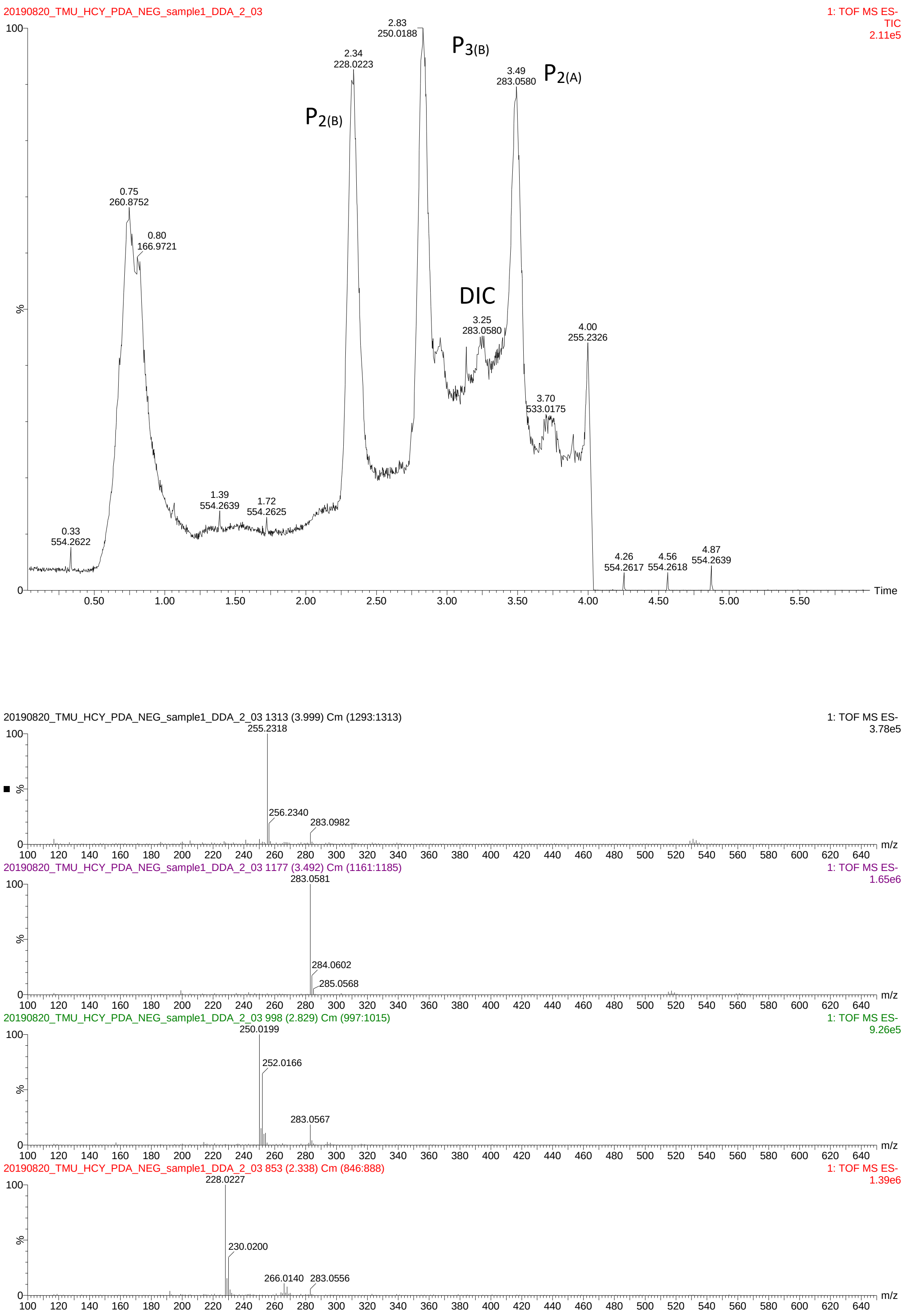

Figure S8. LC/MS (ESI$^-$) chromatographic patterns of degradation intermediates in the presence of CTAB. (pH 5.0, reaction time = 2 h, $[MnO_2]_0$ = 400 mg L^{-1}).
